# A score to predict *Pseudomonas aeruginosa* infection in older patients with community-acquired pneumonia

**DOI:** 10.1186/s12879-023-08688-w

**Published:** 2023-10-19

**Authors:** Kingkarn Wijit, Paveena Sonthisombat, Jaruwan Diewsurin

**Affiliations:** 1 The College of Pharmacotherapy of Thailand, Nonthaburi, Thailand; 2 Department of Pharmacy Practice, Faculty of Pharmaceutical Sciences, Naresuan University, Phitsanulok, Thailand; 3 Department of Medicine, Buddhachinaraj Hospital, Phitsanulok, Thailand

**Keywords:** Community-acquired pneumonia, Elderly, *P. aeruginosa*, Predictive risk score

## Abstract

**Background:**

In Thailand, the incidence of community-acquired pseudomonal pneumonia among 60- to 65-year-olds ranges from 10.90% to 15.51%, with a mortality rate of up to 19.00%. Antipseudomonal agents should be selected as an empirical treatment for elderly patients at high risk for developing this infection. The purpose of this study was to identify risk factors and develop a risk predictor for *Pseudomonas aeruginosa* infection in older adults with community-acquired pneumonia (CAP).

**Methods:**

A retrospective data collection from an electronic database involved the elderly hospitalized patients with *P. aeruginosa*- and non-*P. aeruginosa*-causing CAP, admitted between January 1, 2016, and June 30, 2021. Risk factors for *P. aeruginosa* infection were analysed using logistic regression, and the instrument was developed by scoring each risk factor based on the beta coefficient and evaluating discrimination and calibration using the area under the receiver operating characteristic curve (AuROC) and observed versus predicted probability (E/O) ratio.

**Results:**

The inclusion criteria were met by 81 and 104 elderly patients diagnosed with CAP caused by *P. aeruginosa* and non-*P. aeruginosa*, respectively. Nasogastric (NG) tube feeding (odd ratios; OR = 40.68), bronchiectasis (B) (OR = 4.13), immunocompromised condition (I) (OR = 3.76), and other chronic respiratory illnesses (r) such as atelectasis, pulmonary fibrosis, and lung bleb (OR = 2.61) were the specific risk factors for infection with *P. aeruginosa*. The “60-B-r-I-NG” risk score was named after the 4 abbreviated risk variables and found to have good predicative capability (AuROC = 0.77) and accuracy comparable to or near true *P. aeruginosa* infection (E/O = 1). People who scored at least two should receive empirically antipseudomonal medication.

**Conclusions:**

NG tube feeding before admission, bronchiectasis, immunocompromisation, atelectasis, pulmonary fibrosis and lung bleb were risk factors for pseudomonal CAP in the elderly. The 60-B-r-I-NG was developed for predicting *P. aeruginosa* infection with a high degree of accuracy, equal to or comparable to the existing *P. aeruginosa* infection. Antipseudomonal agents may be started in patients who are at least 60 years old and have a score of at least 2 in order to lower mortality and promote the appropriate use of these medications.

## Background

Community-acquired pneumonia (CAP) is lung infection that started outside the hospital or within 48 h of hospital admission [1]. It is a prevalent infectious disease affecting the elderly, with more than 30–40% of cases hospitalized [2]. *Klebsiella pneumoniae* (20.4%), *P. aeruginosa* (15.5%), methicillin-susceptible *Staphylococcus aureus* (8.9%), *Acinetobacter baumannii* (7.3%), *Haemophilus parainfluenzae* (6.7%), *H. influenzae* (5.9%), and *Streptococcus pneumoniae* (5.9%) were identified as the causative agents of CAP in Thai patients aged 60 or older [3]. In addition, 60.80% of *P. aeruginosa* lung infections were determined to be severe, which was twice as high as for other pathogens. Compared to other pathogens’ mortality rate of 5.5%, the 30-day mortality rate associated with *P. aeruginosa* infections was statistically substantially higher (18.20%), and the risk of 30-day mortality was 2.40 times higher than that of others [4].

A review of the literature revealed substantial numbers of risk factors for *P. aeruginosa* infection in elderly patients including previous *P. aeruginosa* infection or colonization within the past year (OR 16.10–19.20), enteral tube feeding (OR 13.90), tracheostomy (OR 6.50–6.95), bronchiectasis (OR 2.90–6.10), male (OR 3.71), lung abscess or empyema (OR 3.4), chronic obstructive pulmonary disease (COPD) (OR 1.8–2.89), indwelling catheters, including central venous and bladder catheters (OR 2.49), other chronic respiratory diseases (OR 2.30), and use of inhaled corticosteroids (OR 1.76) [4–9].

To help reduce mortality from CAP caused by *P. aeruginosa* infection, infection risk prediction tools are therefore essential. Currently, there is no specific predictive tool for assessing the infection risk of *P. aeruginosa* in elderly CAP patients.

This study was conducted to identify risk factors and develop a predictive tool for the risk of CAP caused by *P. aeruginosa* in the elderly in order to develop a guideline for the use of antipseudomonal agents as empiric treatment.

## Methods

### Study design and setting

An analysis of older patients with CAP admitted to a single general hospital with 1,000 beds between January 1, 2016, and June 30, 2021, was done retrospectively. CAP was identified according to the International Classification of Diseases, 10th Revision (ICD-10).

### Inclusion and exclusion criteria

Eligible patients were at least 60 years old, had pneumonia with new pulmonary infiltration on chest radiograph and respiratory symptoms including fever, coughing, or pleuritic chest pain, had not been hospitalized within the previous 14 days, and had a pathogen identified from sputum culture. Patients who had pneumonia together with other organ infections (apart from bloodstream infections) or who lacked information needed to determine risk factors were excluded from the study.

### Definitions

Elderly were defined as patients were at least 60 years old according to Thai criteria.

Chronic respiratory diseases were categorized into three groups: COPD, bronchiectasis, and others.

Immunocompromised status was defined as: receiving prednisone greater than 15 mg/day for longer than 2 weeks or stopped within the past 2 weeks; having a neutrophil count of 500/mm^3^ or less; being on immunosuppressive therapy; presenting with active solid organ/haematologic malignancies or receiving chemotherapy within the past 6 months; having an human immunodeficiency virus (HIV) infection with a CD4+ lymphocyte count of less than 200/µL; or having received a solid organ or hematopoietic stem cell transplant within the previous year.

Severe CAP was defined according to the criteria outlined by the Infectious Diseases Society of America (IDSA) and The American Thoracic Society (ATS) as follows: having at least one major criterion (invasive mechanical ventilation; septic shock requiring vasopressors) or having three of the minor criteria (a respiratory rate of at least 30 breaths/min; a PaO_2_/FiO_2_ ratio of no more than 250; multilobar infiltrates; confusion/disorientation; uremia with a BUN level of at least 20 mg/dL; leukopenia with a WBC count less than 4,000 cells/mm^3^; thrombocytopenia with a platelet count less than 100,000 cells/mm^3^; hypothermia with a core temperature less than 36°C; and hypotension requiring aggressive fluid resuscitation).

Malnutrition was defined as having a BMI of less than 18 kg/m^2^.

### Data collection

Baseline patient characteristics, microbiological findings, and factors for risk factors for *P. aeruginosa* infection were gathered from the electronic Hospital Information System database, which is used in hospitals across Thailand. The data was collected using a spreadsheet by a single researcher.

### Sputum collection methods

Standard microbiological methods were used to analyze the pathogens in sputum. Sputum from the patient’s respiratory tract was obtained through coughing or suction, and ward nurses who had received training in the practical guidelines for collecting specimens for the diagnosis of respiratory tract infections collected it into sterile containers and immediately transported it to the microbiology laboratory. Good-quality sputum samples (with more than 25 polymorphonuclear cells and fewer than 10 epithelial cells per low-power field at a total magnification of × 100) were cultured sequentially on chocolate agar, sheep blood agar, and MacConkey agar. Pathogens were identified using biochemical tests or an automated system.

### Statistical analysis

All the data were analysed using version 14.1 of STATA (StataCorp LLC, College Station, Texas, USA). Depending on the data type, patients’ characteristics were presented as frequency and percentage, mean ± SD or median, and interval range (IQR). Baseline characteristics between the patients with pseudomonal and non-pseudomonal CAP were compared using the chi-squared or Fisher’s exact test. Our research employed a two-sided alpha error of 0.05, so a *p*-value less than 0.05 is statistically significant.

During model development, relevant independent variables (*p*-value < 0.20) were incorporated using bivariate selection. As *P. aeruginosa* infection was the anticipated outcome, multivariable logistic regression was employed, and the OR and 95% confidence interval (95%CI) were reported. We conducted a complete-case analysis on every result.

### Constructing a risk prediction model

#### Assignment of weights to each risk factor

According to Sullivan et al. [10], the beta coefficients for each risk factor in the multivariable logistic regression analysis were calculated to provide a point-based system. The constant (C), which matched the beta coefficient of the risk factor with the lowest value, was then established. Then, in order to assign scores to each risk factor, the beta coefficient of each additional risk factor was divided by the magnitude of C.

#### Model performance

The area under the receiver operating characteristic curve (AuROC), a comparison of true positive rate (TPR) indicating the presence of *P. aeruginosa* and the false positive rate (FPR) indicating the absence of *P. aeruginosa* as the criterion changes, was used to evaluate discrimination, where an AuROC of less than 0.500, 0.500–0.699, 0.700–0.799, 0.800–0.899, and at least 0.900 indicates no, low, good, very good, and excellent predictive power, respectively.

E/O represents the ratio between the expected and observed number of events, with E/O less than 1.00, equal to 1.00, and greater than 1.00 indicating that the model predicts that the incidence of *P. aeruginosa* infection is less than, equal to or nearly equal to, and greater than the actual incidence, respectively.

$$\begin{array}{c}Sensitivity\,\left(Se\right)=\frac{TP}{TP\;+\;FN}\\Specificity\,\left(Sp\right)=\frac{TN}{FP\;+\;TN}\\\begin{array}{c}Positive\,predictive\,value\left(PPV\right)=\frac{TP}{TP\;+\;FP}\\Negative\,predictive\,value\left(NPV\right)=\frac{TN}{TN\;+\;FN}\end{array}\end{array}$$

- Likelihood ratio for a positive result (LR+)
  $$LR+ = \frac{Sensitivity}{1-Specitivity}$$
- Likelihood ratio for a negative result (LR-)
  $$LR- = \frac{1-Sensitivity}{Specitivity}$$
- Accuracy (proportional of correctly classified subjects, CCR)
  $$CCR=\left(\frac{TP+TN}{TP+FP+TN+FN}\right)\times 100$$
- TP, true positive; FP, false positive; FN, false negative; TN, true negative

#### Internal validation

The bootstrap validation method was used for an internal validation [11]. We ran 500 cycles of bootstrapping and calculated the average optimism as the difference between the bootstrap and test performance in terms of AuROC, calibration-in-the-large, and calibration slope. To retrieve the optimism-corrected model, we utilized the uniform shrinkage method and carried out the following three steps: first, a constant term was subtracted from the linear predictor. Then, all coefficient values were multiplied by the optimism-adjusted AuROC derived from the internal validation. The new constant term was finally recalculated. In addition, we created a calibration plot based on the optimism-adjusted model.

#### Refitting method

The Cslope derived from internal validation was utilized to calculate the tool’s intercept. AuROC and E/O were then used to evaluate the predictive power of the tool and the accuracy of *P. aeruginosa* infection, respectively.

#### Optimal cut-off point selection

The issue was the selection of a cut-off point with adequate sensitivity and specificity for predicting *P. aeruginosa* infection risk using the Youden index (J) method. An appropriate threshold for the instrument used to predict the risk of *P. aeruginosa* infection [J = sensitivity − (1 − specificity)] or consider the probability of *P. aeruginosa* infection for each tool score.

## Results

Among 185 senior CAP patients, 81 (43.78%) had *P. aeruginosa* identified from sputum cultures, whereas 104 of them had infection from other pathogens, including *Klebsiella pneumoniae* (56.73%), *Streptococcus pneumoniae* (19.23%), *Haemophilus influenzae* (8.65%), *Staphylococcus aureus* (5.77%), *Escherichia coli* (5.77%), and others (3.85%). Significantly more frequently than in the latter group, the *P. aeruginosa* group displayed the following characteristics: bronchiectasis, other chronic respiratory diseases, including atelectasis, pulmonary fibrosis, and lung bleb, immunosuppressed condition (those who have receiving prednisone greater than 15 mg/day for longer than 2 weeks or stopped within the past 2 weeks, having neutrophil count less than 500/mm^3^, being on immunosuppressive therapy, presenting with active solid organ/haematologic malignancies or receiving chemotherapy within the past 6 months, and having human immunodeficiency virus (HIV) infection with a CD4+ lymphocyte count of less than 200/µL were 5, 2, 24, and 2 cases, respectively), tracheostomy or NG tube feeding prior to hospitalization, use of a ventilator or intravenous antimicrobial therapy, or hospitalization within the previous 3 months (Table 1). For this study, the impact of receiving oral corticosteroids on *P. aeruginosa* infection was not investigated due to the limited number of patients, as previously mentioned.

**Table 1:** Characteristics of elderly patients with CAP

**Patient characteristics**	**Number of patients (%)**		***P*-value**
	***P. aeruginosa* group (*n* = 81)**	**Non-*P. aeruginosa* group (*n* = 104)**	
Male	50 (61.73)	58 (55.77)	0.415
Severe CAP	55 (67.90)	32 (30.77)	0.000
Chronic obstructive pulmonary diseases	28 (34.57)	25 (24.04)	0.116
Bronchiectasis	14 (17.28)	5 (4.81)	0.007
Chronic respiratory diseases (atelectasis, lung fibrosis, and lung bleb)	26 (32.10)	12 (11.54)	0.001
Tracheostomy before hospitalization	12 (14.81)	2 (1.92)	0.001
Nasogastric tube feeding prior to admission	18 (22.22)	1 (0.96)	0.000
Taking inhaled corticosteroids before hospitalization	21 (25.93)	17 (16.35)	0.142
Receiving intravenous antimicrobials within the past 3 months	29 (35.80)	15 (14.42)	0.001
History of hospitalization within the previous 3 months	40 (49.38)	25 (24.04)	0.001
Use of a ventilator within the past 3 months	13 (16.05)	5 (1.81)	0.013
Receiving proton pump inhibitors before hospitalization	13 (16.05)	21 (20.19)	0.567
Malnutrition	26 (32.10)	20 (19.23)	0.059
Bedridden status	21 (25.93)	16 (15.38)	0.095
Chronic heart failure	5 (6.17)	9 (8.65)	0.587
Cerebrovascular diseases	19 (23.46)	19 (18.27)	0.464
Chronic neurological disorders	15 (18.52)	16 (15.38)	0.692
Chronic kidney diseases (GFR < 35 mL/min/1.73 m^2^)	8 (9.88)	15 (14.42)	0.380
Diabetes mellitus type 2	26 (32.10)	31 (29.81)	0.738
Immunocompromised status	21 (25.93)	8 (7.69)	0.001
Alcohol drinking	1 (1.23)	7 (6.73)	0.081
Smoking	6 (7.41)	9 (8.65)	0.794

As shown in Table 1, patients with *P. aeruginosa* infections exhibited severe symptoms in two-thirds of cases, compared to 30% of patients with non-*P. aeruginosa* infection. The proportion of men, the prevalence of COPD, the use of inhaled corticosteroids or proton pump inhibitors before hospitalization, malnutrition, bedridden patients, chronic heart failure, cerebrovascular disease, chronic neurological disorders, chronic kidney disease with GFR less than 35 mL/min/1.73 m^2^, type 2 diabetes mellitus, alcohol use, or smoking were not statistically different between the two groups.

### Factors associated with *P. aeruginosa* infection in the elderly with CAP

Tracheostomy before hospitalization, use of a ventilator in the last 3 months, receiving intravenous antimicrobials within the past 3 months, history of hospitalization within the past 3 months, malnutrition, taking inhaled corticosteroids before hospitalization, COPD, and alcohol consumption were the major risk factors from univariate logistic regression analysis that were not significant in multivariate logistic regression analysis.

The following elements were shown to still be statistically significant after multivariate logistic regression analysis: NG tube feeding prior to admission, bronchiectasis, immunosuppressed condition, and other chronic respiratory diseases, which include atelectasis, pulmonary fibrosis, and lung bleb. These factors increased the risk of *P. aeruginosa* infection by 40.98 (OR 40.98; 95%CI 3.85–429.33), 4.13 (OR 4.13; 95%CI 1.28–13.34), 3.67 (OR 3.76; 95%CI 1.23–11.51), and 2.61 (OR 2.61; 95%CI 1.05–6.44) times, respectively, when compared to individuals without these characteristics (Table 2).

**Table 2:** Logistic regression analysis of *P. aeruginosa* infection risk variables in elderly individuals with CAP

**Patient characteristics**	**Univariate logistic regression analysis**		**Multivariate logistic regression analysis**	
	**Odds ratio (95%CI)**	***p*-value**	**Odds ratio (95%CI)**	***p*-value**
Male	1.28 (0.71–2.31)	0.415		
Chronic obstructive pulmonary diseases	1.67 (0.88–3.17)	0.117^*^	2.09 (0.80–5.48)	0.133
Bronchiectasis	4.14 (1.42–12.03)	0.005^*^	4.13 (1.28–13.34)	0.018^‡^
Chronic respiratory diseases (atelectasis, pulmonary fibrosis, and lung bleb)	3.62 (1.69–7.76)	0.001^*^	2.61 (1.05–6.44)	0.038^‡^
Tracheostomy before hospitalization	8.87 (1.92–40.87)	0.001^*^	1.13 (0.15–8.51)	0.903
Nasogastric tube feeding prior to admission	29.43 (3.83–225.85)	0.000^*^	40.68 (3.85–429.33)	0.002^‡^
Taking inhaled corticosteroids before hospitalization	1.79 (0.87–3.68)	0.111^*^	1.01 (0.35–2.88)	0.990
Receiving intravenous antimicrobials within the past 3 months	3.31 (1.63–6.74)	0.001^*^	0.90 (0.25–3.24)	0.871
History of hospitalization within the past 3 months	3.08 (1.65–5.77)	0.000^*^	1.63 (0.52–5.11)	0.402
Use of a ventilator in the last 3 months	3.79 (1.29–11.11)	0.010^*^	1.89 (0.44–8.13)	0.393
Receiving proton pump inhibitor before hospitalization	0.76 (0.35–1.62)	0.468		
Malnutrition	1.99 (1.01–3.90)	0.045^*^	1.23 (0.51–2.98)	0.648
Bedridden	1.92 (0.93–3.99)	0.076^*^	0.91 (0.29–2.84)	0.864
Chronic heart failure	0.69 (0.22–2.19)	0.523		
Cerebrovascular diseases	1.37 (0.67–2.80)	0.388		
Chronic neurological disorders	1.25 (0.58–2.71)	0.572		
Chronic kidney disease (GFR < 35 mL/min/1.73 m^2^)	0.65 (0.26–1.62)	0.348		
Diabetes mellitus type 2	1.11 (0.59–2.09)	0.738		
Immunocompromised status	4.20 (1.75–10.08)	0.001^*^	3.76 (1.23–11.51)	0.020^‡^
Alcohol drinking	0.17 (0.02–1.44)	0.050^*^	0.08 (0.00–3.91)	0.201
Smoking	0.84 (0.29–2.48)	0.757		

^*^P < 0.20 for univariate logistic regression analysis

^‡^*P* < 0.05 for multivariate logistic regression analysis

### Constructing a risk prediction model

#### Assignment of weights to each risk factor

**Fig. 1:**
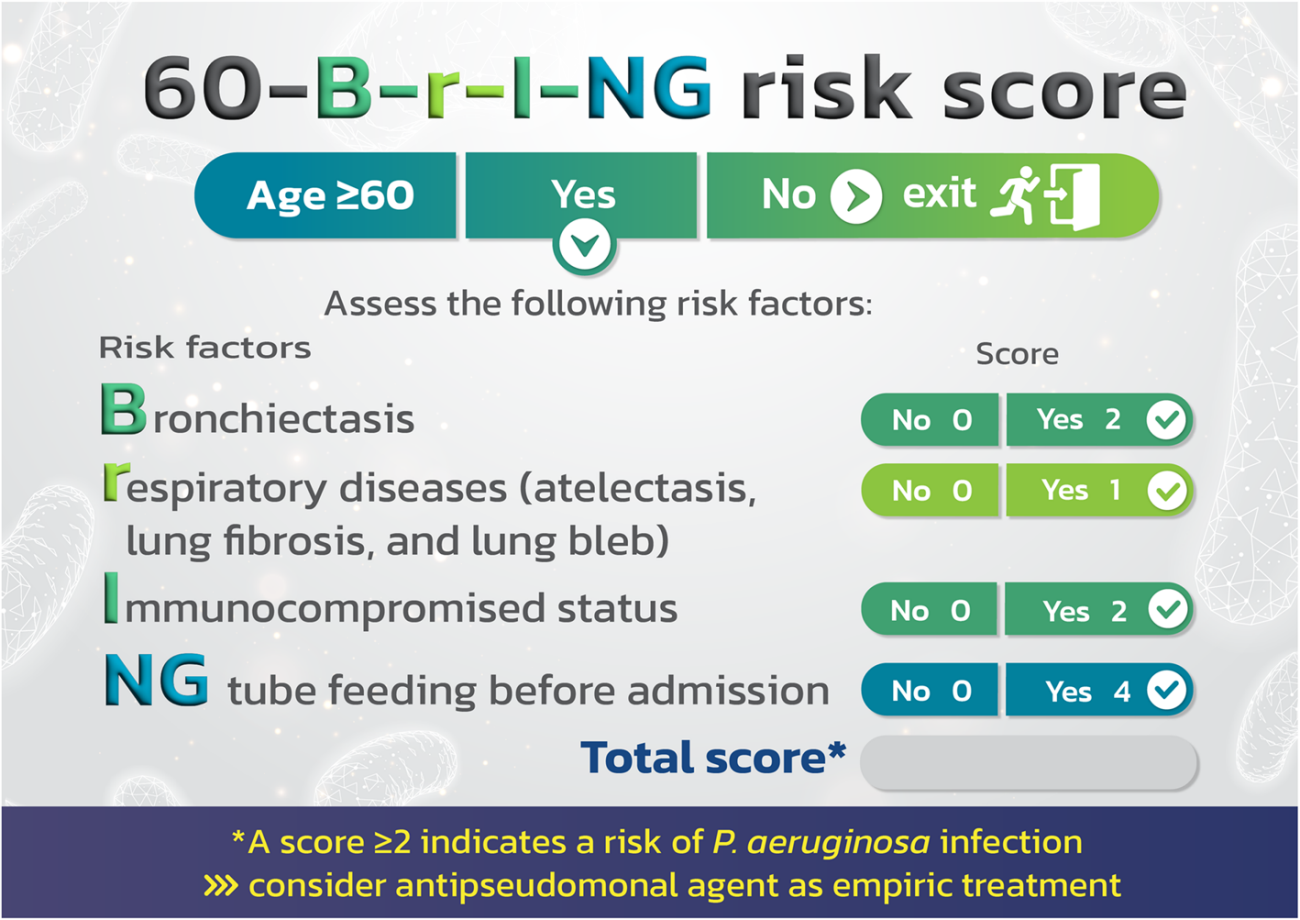
60-B-r-I-NG risk score for predicting the risk of *P. aeruginosa* infection in elderly CAP patients

The beta coefficient for each risk factor was determined. As can be seen in Table 3, the beta coefficients for NG tube feeding before admission (NG), bronchiectasis (B), and immunosuppressed status (I) were divided by the lowest value for other chronic respiratory disease (r) to get scores of 4, 2, and 2 correspondingly. To make the tool more recognizable and to underline that it is used to predict the likelihood of *P. aeruginosa* infection in CAP patients 60 years of age and above, we gave it the name 60-B-r-I-NG risk score (Fig. 1).

**Table 3:** Beta coefficient and score determination of *P. aeruginosa* infection risk factors

**Factors**	**Beta Coefficient**^**a**^	***P*-value**	**Transformed coefficients**	**Assigned score**
Nasogastric tube feeding prior to admission	3.62	0.001	3.93	4
Bronchiectasis	1.86	0.001	2.02	2
Immunocompromised status	1.48	0.003	1.61	2
Other chronic respiratory diseases (atelectasis, pulmonary fibrosis, and lung bleb)	0.92	0.039	1	1

^a^Coefficient of model intercept = −1.140522

#### Model performance

**Fig. 2:**
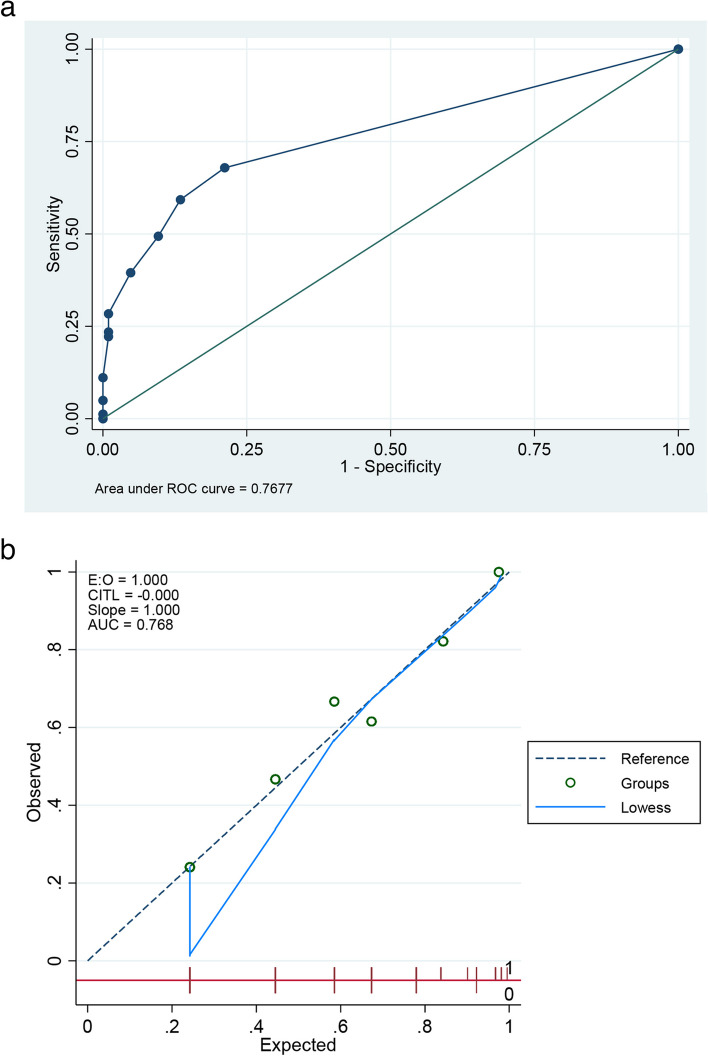
AuROC and calibration curve of 60-B-r-I-NG risk score for *P. aeruginosa* infection prediction. **a** AuROC of the 60-B-r-I-NG risk score. **b** Calibration curve of the 60-B-r-I-NG risk score

With an AuROC of 0.77, the 60-B-r-I-NG risk score had a good ability to predict the presence of *P. aeruginosa* infection (Fig. 2a).

Based on a comparison of the predicted risk of *P. aeruginosa* infection with the observed risk, Fig. 2b demonstrates that the calibration of the 60-B-r-I-NG risk score was at or near the actual incidence of *P. aeruginosa* infection (E/O = 1).

#### Internal validation and refitting method

**Fig. 3:**
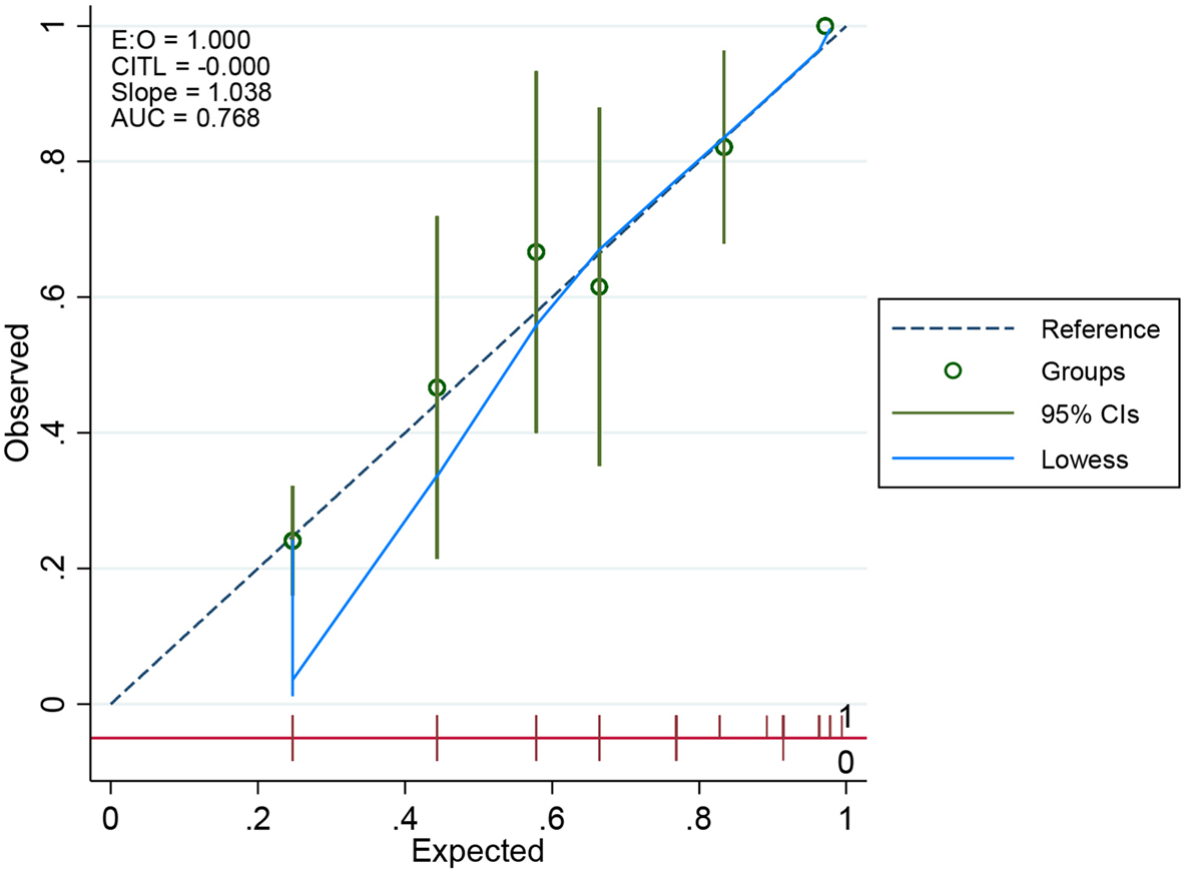
Calibration curve of refitting 60-B-r-I-NG risk score

Using bootstrap validation, the 60-B-r-I-NG risk score for each randomization was compared to the presence of *P. aeruginosa* infection after 500 randomizations of patient data, obtaining a Cslope of 0.96 (not shown), which showed that the proportion of *P. aeruginosa* group and non-*P. aeruginosa* group was similar to that in the original patient group. However, recalibration was carried out to better extrapolate the risk score to other populations by recalculating the intercept and slope based on our data. The Cslope result of 1.038, with an AuROC and E/O remaining the same (0.77 and 1, respectively), showing that this instrument can be applied to other populations (Fig. 3).

#### Optimal cut-off point selection

**Fig. 4:**
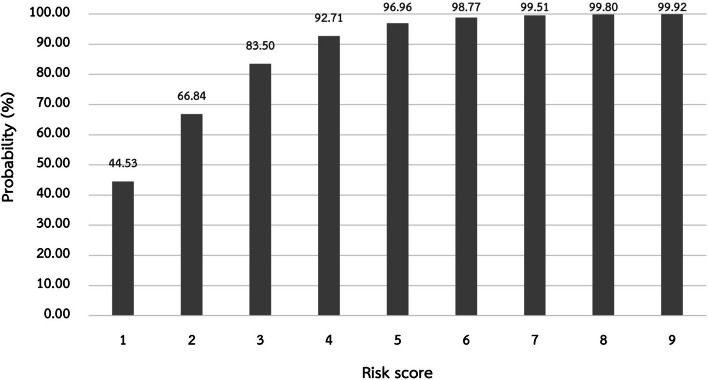
Probability of *P. aeruginosa* infection at various scores of the 60-B-r-I-NG model

Using the 60-B-r-I-NG risk score to predict *P. aeruginosa* infection risk, the optimal score was determined by calculating the sensitivity and specificity of each score using the Youden index. The highest and most comparable Youden index values for the 60-B-r-I-NG risk score were 0.47 and 0.46, with sensitivity and specificity values of 67.90% and 78.85%, and 59.26% and 86.54%, respectively (Table 4). Therefore, the cut-off points 1 or 2 was could be used to predict the risk of *P. aeruginosa* infection. However, taking into account that when the cut-off score was 2 points, the likelihood of *P. aeruginosa* infection (66.84 percent) was higher (Fig. 4). The authors came to the conclusion that a 60-B-r-I-NG risk score of at least 2 suggests a risk for *P. aeruginosa* infection in elderly patients with CAP.

**Table 4:** Ability of 60-B-r-I-NG risk score to predict *P. aeruginosa* infection at different cut-off points

**Score**	**Sensitivity (%)**	**Specificity (%)**	**PPV (%)**	**NPV (%)**	**LR+**	**LR−**	**Accuracy (%)**	**Youden index**
0	100.00	0.00	43.78		1.00		43.78	
1	67.90	78.85	71.43	75.93	3.21	0.41	74.05	0.47
2	59.26	86.54	77.42	73.17	4.40	0.47	74.59	0.46
3	39.51	95.19	86.48	66.90	8.22	0.64	70.81	0.35
4	23.46	99.04	95.01	62.43	24.40	0.78	65.95	0.23
5	11.11	100.00	100.00	59.09		0.89	61.08	0.11
6	4.94	100.00	100.00	57.46		0.95	58.38	0.05
7	1.23	100.00	100.00	56.52		0.99	56.76	0.01
> 7	0.00	100.00		56.22		1.00	56.22	

*LR+* Likelihood ratio for a positive result, *LR-* Likelihood ratio for a negative result, *NPV* Negative predictive value, *PPV* Positive predictive value

## Discussion

Even while *P. aeruginosa* only causes severe CAP in only 1.8% to 8.3% of patients, it contributes to a high mortality rate of 50% to 100% [4]. Therefore, empiric therapy for patients with suspected *P. aeruginosa* infection is necessary to reduce mortality.

According to country-specific treatment guidelines, the risk factors for *P. aeruginosa* pulmonary infection vary. These include severe underlying lung disease, recurrent bronchiectasis, use of antibiotics within the previous 3 months, recent hospitalization, airway *P. aeruginosa* colonization, history of antibiotic therapy for 2 or more days within the previous 90 days, current tube feeding, and alcohol consumption [12–18].

A history of respiratory isolation of *P. aeruginosa* during the previous year and hospitalization with taking parenteral antibiotics within the preceding 90 days are both identified as risk factors for *P. aeruginosa* infection in the most recent 2019 treatment guidelines from the IDSA and ATS [1]. However, they stress that the most crucial ones are locally confirmed risk factors for *P. aeruginosa* infections because prior research from various population and geographic studies has produced inconsistent results about the associations between risk factors and *P. aeruginosa* infections.

The following risk factors were of particular interest: infection with or colonization by *P. aeruginosa* within the past year (OR 16.10; 95%CI 9.48–27.35) [6], enteral tube feeding (OR 13.87; 95%CI 3.39–56.65) [8], tracheostomy (OR 6.50; 95%CI 2.61–16.19) [6], hospitalization for more than 2 days within the past 30 days but not within the past 7 days (OR 3.8; 95%CI 1.8–8.3) [19], male (OR 3.71; 95%CI 1.65–8.35) [4], and immunodeficiency (OR 1.39; 95%CI 1.22–1.58) [20]. Despite definitional differences, the majority of studies have found that bronchial or pulmonary diseases, including asthma, uncomplicated chronic bronchitis, COPD, bronchiectasis, and interstitial lung disease, are risk factors for *P. aeruginosa* infection (OR 1.25–5.8; *p* < 0.05) [4, 6, 8, 19]. In addition, severe COPD or pneumonia necessitating mechanical ventilation or vasopressors, and having a pneumonia severity index (PSI) risk class of IV–V, are considered high risk (OR 1.85–3.95) [4, 6, 20]. Interestingly, recent exposure to inhaled corticosteroids within the past 90 days (OR 1.40; 95%CI 1.23–1.61), receiving Gram-positive coverage therapy within the past 90 days (OR 1.37; 95%CI 1.01–1.87), prior hospitalization within the past 90 days (OR 1.36; 95%CI 1.21–1.54), and use of beta-lactams within the past 90 days (OR 1.31; 95%CI 1.14–1.51) are weakly associated with *P. aeruginosa* infection [19]. One study also discovered that having diabetes (OR 0.82; 95%CI 0.70–0.95) and being older than 84 years old (OR 0.64; 95%CI 0.52–0.78) decreased the likelihood of contracting this pathogen [20].

One benefit of this study was the tool’s ability to precisely examine risk factors for *P. aeruginosa* infection while various predictive scores were developed to evaluate the risk of infection from not only *P. aeruginosa* but also methicillin-resistant *Staphylococcus aureus* (MRSA), extended-spectrum beta-lactamase (ESBL)-producing Enterobacteriaceae, *Stenotrophomonas maltophilia*, enterococci, and *Acinetobacter baumannii*, in patients with community-onset, healthcare-associated pneumonia [21–30]. Although not all of the studies listed in Table 5 provided evidence that our identified risk factors, such as NG tube feeding, bronchiectasis, or immunocompromised, were risk factors for *P. aeruginosa* infection, we believed that our instrument developed based on locally validated risk factors that have been shown to influence *P. aeruginosa* infection in patients over 60 years of age diagnosed with CAP. Another advantage was that only our study found a link between pseudomonal pneumonia and lung bleb, atelectasis, and pulmonary fibrosis.

**Table 5:** Model comparison of risk variables related to particular pathogens

**Risk factors/Scores**	**60-B-r-I-NG risk score**	**The IDSA and ATS 2019 **[1]	**Aliberti score **[21]	**Shorr score **[22]	**Shindo score **[23]	**ARUC score **[24]	**Tree-structured prediction analysis **[25]	**Schreiber score **[26]	**Park score **[27]	**Score to PES **[28]	**Resistant bacterial pneumonia risk score **[29]	**Song tool (HDAP) **[30]
Pathogens	PA	MRSA and PA	MDR	MRSA, PA, and ESBL-E	Drug resistant^a^	MRSA, *S. maltophilia*, ESBL-E or CRE	MRSA and PA	MRSA, PA, and ESBL-E	PDR^b^	PA, ESBL-E, and MRSA	MRSA, PA, and ESBL-E	MDR
NG tube feeding prior to admission	✓				✓				✓			
Bronchiectasis	✓									✓	✓	
Immunocompromised status	✓		✓					✓			✓	
Other chronic respiratory diseases^c^	✓											
Prior respiratory isolation of MRSA or *P. aeruginosa*		✓										
Recent hospitalization (last 90 d)		✓		✓		✓			✓		✓	✓
Receipt of parenteral antibiotics		✓ last 90 d	✓ Last 90 d		✓ Last 90 d	✓ Last 30 d	✓ Last 6 m	✓ Last 30 d	✓ Last 30 d	✓ Last 30 d		
Cerebrovascular disease			✓									
Diabetes			✓									
COPD			✓							✓		
Wound care within 30 d			✓						✓		✓	
Chemotherapy within 30 d									✓			
Home infusion therapy			✓								✓	
Chronic kidney disease			✓							✓		
Nursing home or LTC facility				✓		✓		✓	✓		✓	
Chronic hemodialysis/ chronic dialysis				✓		✓			✓		✓	✓
Admitted to the ICU within 24 h				✓								
Use of gastric acid-suppressive agents					✓							
Nonambulatory status					✓							
Bilateral pulmonary infiltration						✓						
Pleural effusion						✓						
PaO2/FiO2 < 300						✓						
The Activity of Daily Living score							✓					
Age ≥ 40										✓		
Male										✓		
Altered mental status										✓		
Severe pneumonia											✓	
PSI > 147												✓

*COPD* Chronic obstructive pulmonary disease, *CRE* Carbapenem-resistant Enterobacteriaceae, *ESBL-E* Extended spectrum β-lactamases-producing Enterobacteriaceae, *HDAP* Hemodialysis-associated pneumonia, *ICU* Intensive care unit, *LTC* Long-term care, *MDR* Multidrug resistant, *MRSA* Methicillin-resistant *S. aureus*, *NG tube* Nasogastric tube, *PA P. aeruginosa*, *PDR* Potentially drug-resistant, *PSI* Pneumonia severity index

^a^Methicillin-resistant *S. aureus*, *P. aeruginosa*, and ESBL-producing Enterobacteriaceae that were not susceptible to beta-lactams (ceftriaxone or ampicillin-sulbactam), macrolides (azithromycin or clarithromycin), and fluoroquinolones (moxifloxacin, levofloxacin, or garenoxacin)

^b^Methicillin-resistant *S. aureus*, *P. aeruginosa*, *A. baumannii*, *Stenotrophomonas maltophilia*, and ESBL-producing Enterobacteriaceae

^c^Atelectasis, pulmonary fibrosis, and lung bleb

This study identified NG tube feeding prior to admission as the strongest predictor of *P. aeruginosa* infection. The organism can be found in the environment, particularly in water. After entering the body via the respiratory tract, biofilms are easily formed on the inner surface of tubes [31]. In one study, *P. aeruginosa* was cultured from the tongue dorsal swabs of 34% of elderly patients who wore NG tubes for at least 2 weeks, whereas no such bacteria were found in the group without NG tubes (50 cases; *p* < 0.001). Scanning electron micrography analysis of samples from the oropharyngeal section of the NG tube revealed that the biofilm was produced by the same strain of *P. aeruginosa* found in the oropharynx of *P. aeruginosa*-infected patients [32]. NG tube feeding being a significant risk factor for *P. aeruginosa* is thus not surprising.

Due to primary antibody deficiencies, bronchiectasis is a risk factor for the development of CAP [33], and *P. aeruginosa* is the leading cause of this disease. When it binds to the airway epithelium with its flagella and pili [34], *P. aeruginosa* secretes various virulence factors that promote cell adhesion and tissue invasion, inhibit mucociliary function, and dysregulate host immunity, leading to airway inflammation and tissue damage. With bronchiectasis, infection and inflammation occur simultaneously in the trachea. This creates favourable conditions for the colonization of pathogens, particularly *P. aeruginosa* [35], through biofilm formation [34], with the severity of inflammation and the amount of colonization correlated with the severity and frequency of bronchiectasis exacerbations [35].

Immunocompromised patients, particularly those with neutrophil counts less than 500/mm^3^, haematologic malignancies, transplant recipients, and HIV infection, are at increased risk for *P. aeruginosa* infection of the pulmonary and circulatory system [34, 36, 37] due to loss of mucosal barriers, mucositis from chemotherapy, and selective pressure from broad-spectrum antimicrobial therapy [36].

The majority of the research participants with *P. aeruginosa* infection experienced atelectasis (73.08%). This anomaly encourages the production of biofilms, making it a risk factor for *P. aeruginosa* infection.

COPD prevalence did not differ between the *P. aeruginosa* and non-*P. aeruginosa* groups, ruling out a link between COPD and *P. aeruginosa* infection in this study. *P. aeruginosa* is typically detected in the sputum of 4% to 15% of COPD patients without a pulmonary infection. In the lungs of COPD patients, there are two types of colonization: short-term colonization followed by eradication and long-term persistence [38]. COPD patients with pulmonary *P. aeruginosa* infection are associated with a higher incidence of acute COPD exacerbations (AECOPD). It also causes chronic infections in the aforementioned patients. Studies indicated that *P. aeruginosa* infection can persist in the lungs of COPD patients for up to a year. Compared to bloodstream isolates from non-AECOPD patients, respiratory samples from AECOPD patients tend to have lower cytotoxicity and motility but produce more biofilm in chronic infections [39].

This study was unable to establish a correlation between a previous infection or colonization with *P. aeruginosa* and the risk of developing a *P. aeruginosa* infection. As this factor was present in only 9 of 185 patients (4.86%) and all cases were infected with *P. aeruginosa*, it was not possible to calculate the OR for comparing the presence of these risk factors for infection with *P. aeruginosa* or other pathogens.

This study has limitations due to the relatively small number of participants, primarily because a high percentage (44.90%) of patients diagnosed with pneumonia in our setting had no bacterial growth in sputum culture. This result was in line with those of a retrospective cohort research carried out at a university hospital in Thailand, which revealed that no bacteria were discovered in sputum cultures 55.15 percent of the time [40]. Furthermore, it was observed that some patients who were initially included in the study were later excluded due to concomitant infections in other organs or insufficient data for identifying risk factors, accounting for 22.00% and 4.00%, respectively.

**Fig. 5:**
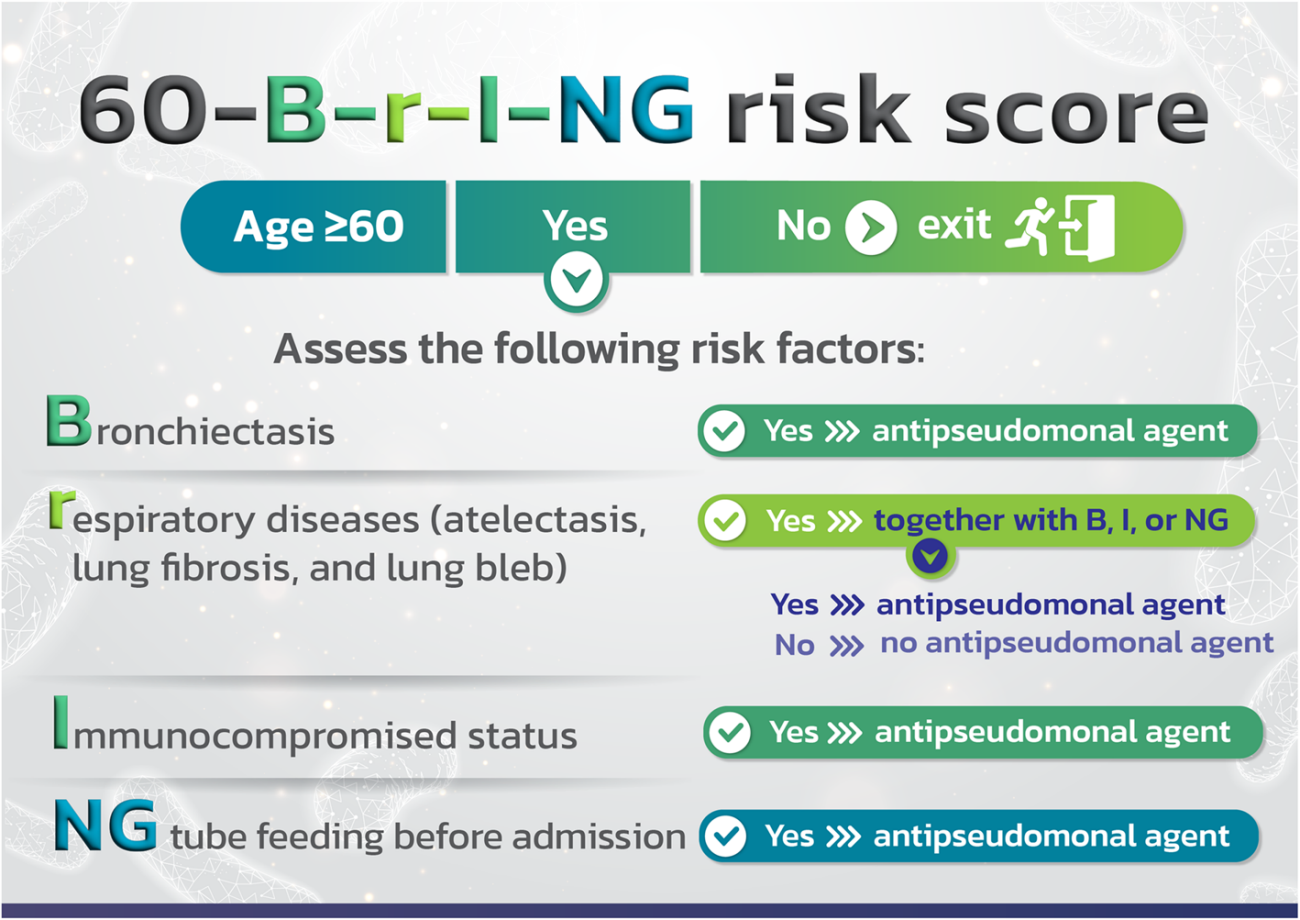
The risk score to the 60-B-r-I-NG checklist

We allocated scores of 4, 2, 2, and 1 for NG tube feeding prior to admission, bronchiectasis, immunosuppressed state, and other chronic respiratory disease, respectively, nevertheless, the cut-off score for the risk of *P. aeruginosa* infection was only 2 points. With the exception of having atelectasis, pulmonary fibrosis, and lung bleb, patients are at risk for contracting *P. aeruginosa* infection even if they only have one risk factor. Therefore, we changed the risk score to the 60-B-r-I-NG checklist (as shown in Fig. 5). CAP cases with NG tube feeding, bronchiectasis, and immunocompromised status should receive empirically antipseudomonal agent based on local susceptibility. If they merely have atelectasis, pulmonary fibrosis, or lung bleb, they do not require antipseudomonal agent.

## Conclusions

According to multivariate logistic regression, NG tube feeding prior to admission, bronchiectasis, immunocompromised status, atelectasis, pulmonary fibrosis, or lung bleb were risk factors associated with *P. aeruginosa* infection in older adults with CAP. The risk score 60-B-r-I-NG was created, and it was discovered to have a high level of prediction power and accuracy, on par with a true *P. aeruginosa* infection. Elderly CAP patients with a risk score of 2 points or above should have empirical antipseudomonal agent treatment, according to the assessment of the 60-B-r-I-NG risk score.

## Data Availability

All the data supporting our findings are contained within the manuscript.

## Abbreviations

AECOPD: Acute chronic obstructive pulmonary disease exacerbations
AUROC: Area under the receiver operating curve
B-r-I-NG: Bronchiectasis, respiratory disease, immunocompromised patients, and nasogastric tube feeding
CAP: Community-acquired pneumonia
COPD: Chronic obstructive pulmonary disease
E/O: Observed versus predicted probability
HIV: Human immunodeficiency virus
NG: Nasogastric
*P. aeruginosa*: *Pseudomonas aeruginosa*
